# A panoramic study of circulating tumor DNA in diffuse large B-cell lymphoma

**DOI:** 10.1016/j.gendis.2026.102038

**Published:** 2026-01-13

**Authors:** Ruoyun He, Wanyue Zhang, Lisha Li, Lintao Bi

**Affiliations:** a Department of Hematology, China-Japan Union Hospital of Jilin University, Changchun, Jilin 130033, China; b The Second Hospital of Jilin University, Changchun, Jilin 130021, China; c The Key Laboratory of Pathobiology, Ministry of Education, College of Basic Medical Sciences, Jilin University, Changchun, Jilin 130021, China

**Keywords:** Circulating tumor DNA, Diffuse large B-cell lymphoma, Dynamicmonitoring, Liquid biopsy, Prognostic evaluation

## Abstract

Diffuse large B-cell lymphoma (DLBCL) is a frequently encountered aggressive form of non-Hodgkin's lymphoma, characterized by significant clinical and molecular heterogeneity. Owing to inherent limitations of traditional treatments for DLBCL, the advent of circulating tumor DNA (ctDNA) detection, coupled with the integration of multi-omics technologies, has brought about substantial advancements in the diagnosis and treatment of this disease. This review elaborates on the evolution of ctDNA detection and multi-omics integration, its applications in early diagnosis, prognostic model construction, and treatment decision-making, as well as drug resistance mechanisms, highlighting that ctDNA is a powerful tool but faces technical, biological, and clinical implementation barriers that need to be overcome for wider clinical use.

## Introduction

Diffuse large B-cell lymphoma (DLBCL) is the most aggressive form of non-Hodgkin's lymphoma, with significant clinical and molecular heterogeneity. In anatomical classifications, primary central nervous system lymphoma and mediastinal lymphoma show significantly different prognosis compared to lymphomas at other sites.^1^ Therefore, this study focuses on diffuse large B-cell lymphoma, not otherwise specified (DLBCL, NOS). The R–CHOP regimen is the standard first-line immunochemotherapy for DLBCL, comprising rituximab, cyclophosphamide, doxorubicin, vincristine, and prednisone. Currently, a substantial subset (30%–40%) of patients treated with this regimen either fail initial therapy or eventually relapse.^2^^,^^3^

Traditional detection methods, such as conventional imaging and invasive tissue biopsies, have long been the cornerstone of monitoring and diagnosing DLBCL. However, these methods have significant limitations, especially in the detection of molecular residual disease (MRD). MRD is a crucial factor in predicting the recurrence of DLBCL and the outcome of treatment, but traditional methods (bone marrow puncture or morphological assessment) have a sensitivity of only 10^−3^ to 10^−4^, which is much lower than that of the new generation of technologies.^4^ This makes it easy to miss detecting residual lymphoma cells. Moreover, tissue biopsy is invasive, accompanied by surgical risks such as bleeding and infection, and it cannot be repeatedly sampled frequently, limiting the feasibility of dynamic monitoring.^5^ Therefore, traditional approaches have obvious deficiencies in reliably identifying MRD in DLBCL, and more sensitive and minimally invasive alternative strategies are needed.^6^ Liquid biopsy, particularly through circulating tumor DNA (ctDNA) profiling, has emerged as a new approach to addressing these diagnostic challenges. This approach deciphers tumor-derived DNA fragments circulating in the bloodstream, providing a dynamic window into real-time tumor burden fluctuations, molecular signature analysis, and evolutionary patterns.^7^, ^8^, ^9^, ^10^ CAPP-Seq achieves unprecedented sensitivity in detecting rare mutations through deep targeted capture and molecular barcoding noise reduction. PhasED-Seq, on the other hand, further filters out background using haplotype phase information. These cutting-edge omics platforms are now achieving unprecedented sensitivity in detecting rare mutations, and in some cases can identify mutations with variant allele frequencies as low as 0.01%.^10^^,^^11^ Together with fragment size profiling and epigenetic mapping, these methods precisely trace the cellular origins of ctDNA while distinguishing tumor-specific signals from background noise.^12^ These advancements have collectively expanded the role of ctDNA in the management of DLBCL. It has evolved from merely detecting mutations to becoming a multifaceted tool. As an auxiliary tool, ctDNA can be used for molecular classification to guide treatment selection, identify MRD, and, in combination with other technologies, assist in assessing disease prognosis and risk stratification, thereby refining therapeutic strategies. It enables risk-based prediction of disease course, tracks treatment response at the molecular level, and characterizes resistance patterns at relapse.^13^^,^^14^

This review outlines ctDNA's role in DLBCL management through three pillars: relapse prevention through ctDNA analysis, resistance mapping via single cell ctDNA sequencing, and immune monitoring with multi-analyte profiling. We propose an integrated framework leveraging ctDNA dynamics for decision-making across treatment phases—from molecular profiling to MRD-guided interventions—while urging cross-sector collaboration to address technical and operational barriers.

## Evolution of ctDNA detection and multi-omics integration

The common ctDNA detection methods shown in Table 1 compares traditional and emerging ctDNA detection technologies in DLBCL.

**Table 1:** Comparison of Traditional and Emerging ctDNA Detection Technologies in DLBCL.

Technology	LOD	Key Advantage	Application	Ref.
ddPCR	1%	Cost-effective for known mutations	Validation of MYD88 L265P	8
CAPP-Seq	0.01%	Deep sequencing of BCL2/MYD88 hotspots	MRD monitoring	10,13
PhasED-Seq	0.001%	Phase-enhanced error correction	Ultra-early relapse detection	3,9
COMOS model	N/A	Integrates fragmentomics/epigenetics	Tissue-of-origin analysis	12

Notes, tDNA, circulating tumor DNA; DLBCL, diffuse large B-cell lymphoma; LOD, limit of detection; ddPCR, droplet digital PCR; CAPP-Seq, cancer personalized profiling by deep sequencing; PhasED-Seq, phase-enhanced error correction sequencing; MRD, minimal residual disease; COMOS, circulating tumor DNA fragmentomics and epigenetics-based Model; N/A, not applicable; CAR T-Cell Therapy, chimeric antigen receptor T-Cell therapy; NGS, next generation sequencing.

In ctDNA detection, DdPCR, as a PCR-based method, is known for its low cost and simple operation but is limited to known mutations, restricting its application in comprehensive screening.^15^ In contrast, amplicon-based NGS offers higher sensitivity and speed, making it suitable for integration into routine diagnostics,^16^ but its targeted nature might miss mutations outside predefined regions.^17^ Hybrid capture-based NGS enables broader mutation detection through probe capture without the need for prior knowledge.^17^

In recent years, innovative methods like PhasED-Seq and CAPP-Seq have further advanced the boundaries of ctDNA detection. CAPP-Seq is effective for baseline analysis with high sensitivity, but its reliance on single mutations limits sensitivity at very low tumor fractions, potentially missing 25% of DLBCL recurrences.^18^ PhasED-Seq, by detecting phased variants, excels in error suppression and recovery efficiency, particularly for DLBCL where clustered mutations exist due to somatic hypermutation.^18^^,^^19^ For instance, in a study by Kurtz et al,^18^ PhasED-Seq successfully identified ctDNA in samples where CAPP-Seq yielded negative results, significantly improving recurrence prediction and even detecting MRD up to 10 months before clinical recurrence, whereas traditional PET-CT failed to do so.^18^

Overall, selecting a ctDNA detection method requires balancing sensitivity, cost, and application scenario. DdPCR is suitable for low-cost screening of known mutations, while NGS methods provide more comprehensive analysis; PhasED-Seq and CAPP-Seq show potential in ultra-sensitive monitoring, especially for MRD assessment. However, even the combined use of these single technologies struggles to address DLBCL's unique challenges, including background noise from non-tumor cfDNA, inability to link mutations to treatment response, and late detection of immunochemotherapy resistance. This limitation highlights the need for a more integrated technical solution. However, there are still two remaining challenges: low ctDNA shedding in double-hit lymphoma (median tumor fraction 7%) requiring cfRNA co-analysis^20^ and lack of standardized variant allele frequencies (VAFs) thresholds across platforms (e.g., ddPCR *vs* NGS).

## Relapse prevention through ctDNA analysis in DLBCL

### Early diagnosis and molecular subtyping

Emerging evidence reveals distinct molecular landscapes in ctDNA across DLBCL subtypes, with baseline ctDNA load serving as a biological mirror reflecting cell-of-origin classifications (Table 2)^21^, ^22^, ^23^. GCB subtypes exhibit a higher median VAF (15.2% *vs* 8.7%, *p =* 0.003) with KMT2D/CREBBP mutations, while ABC ctDNA is NF-κB-driven (MYD88/CD79B mutations correlate with IL-10, r = 0.51).^24^^,^^25^ It is worth noting that compared with the traditional tissue biopsy group, the double-hit lymphoma cases had a better detection effect on MYC/BCL2/BCL6 rearrangements in free DNA (sensitivity of 92% *vs* 78%), and the 2-year progression-free survival rate of patients with positive rearrangements dropped sharply (31% *vs* 68%, *p* < 0.001); liquid biopsy may help bridge the prognosis gap of early intervention.^26^

**Table 2:** Genetic and biomarker characteristics of DLBCL subtypes.

DLBCL Subtype	Related Genes	Biomarkers	Sensitivity	Specificity	Ref.
MCD Subtype	KMT2D	MYD88/CD79B mutations IL-10 (r = 0.51)	92%	78%	21
	CREBBP				
	MYD88				
	CD79B				
ABC Subtype	NF-κB-driven	MYD88/CD79B mutations IL-10 (r = 0.51)	95.65% (CR), 4.35% (PR)	91.30% (CR), 4.35% (PR)	21
	MYD88				
	CD79B				
GCB Subtype	KMT2D	Median VAF 15.2%	82.6% (Non-GCB)	37.7% (GCB)	21
	CREBBP				
Double-hit lymphoma	MYC	Rearrangements	92%	78%	21
	BCL2				
	BCL6				
IVLBCL	MYD88	MYD88 (56%)	85.3% (IPI >3)	73.5% (ECOG ≥ 2)	22
	CD79B	CD79B (44%)			
	TNFAIP3	TNFAIP3 (38%)			
	IRF4	IRF4 (29%)			
Less-defined Subtype	FLT3	PCDI score	4.4 (HR), 3.5–5.6 (95% CI)	1.7 (HR) 1.1–2.7 (95% CI)	23
	SORL1				
	CD8A				
	BCL2L11				
	COL13A1				
	MPG				
	DYRK2				
	CAMK2B				

Notes: DLBCL, diffuse large B cell lymphoma; MCD, myeloid cell differentiation; ABC, activated B cell; GCB, germin B cell; IPI, international prognostic index; IVLBCL, intravascular large B cell lymphoma; PCDI, programmed cell death index.

Additionally, a study conducted in Chinese patients demonstrated the utility of ctDNA in determining cell origin and predicting survival in DLBCL.^27^ The consistency between plasma genotyping classification and traditional cell origin classification using tumor tissue was found to be 80.0% (20/25). Besides, among the 24 patients whose tissue biopsies and ctDNA were assigned to specific clusters using the LymphGen tool, the concordance rate between ctDNA and tissue biopsy was 95.8%.^9^ Moreover, patients with higher baseline plasma ctDNA levels had almost half of the survival rates, indicating the value of pre-treatment ctDNA analysis in clinical origin determination and prognosis prediction.

Moreover, 5hmC alterations in ctDNA reflected molecular differences in DLBCL at various primary sites.^28^ By using the hmC-CATCH technology, the 5hmC profile of plasma cfDNA can accurately distinguish different primary sites (such as lymph nodes, gastrointestinal tract, central nervous system, testicles or bone marrow) of DLBCL with a 92% accuracy rate before treatment. This provides a truly minimally invasive tool for site tracing for patients who cannot undergo biopsy or require repeated monitoring.^28^

### Prognostic model construction and dynamic risk stratification

The integration of ctDNA metrics with established clinical and radiographic indices has significantly advanced the prognostic stratification of DLBCL patients across various treatment modalities. In patients receiving CAR-T cell therapy, early molecular responses are highly predictive of outcomes. For instance, after axicabtagene ciloleucel (axi-cel) infusion, achieving undetectable ctDNA as early as one week strongly correlates with durable responses, while detectable ctDNA on day 28 identifies patients with markedly inferior median progression-free survival (3 months *vs* not reached) and overall survival (19 months *vs* not reached).^29^ Furthermore, specific ctDNA thresholds, both pre-infusion and at week 4 (such as the failure to achieve a major molecular response, MMR), have been validated as robust determinants of treatment failure and shorter event-free survival.^30^

This prognostic power extends to conventional immunochemotherapy. For newly diagnosed DLBCL, the combination of end-of-treatment (EOT) ctDNA status and the International Prognostic Index (IPI) powerfully discriminates risk; patients with EOT ctDNA-negative status and IPI ≤ 3 achieve a 2-year overall survival of 94%, compared to only 28% in those who are ctDNA-positive with IPI > 3^31^. Moreover, complementary with PET/CT, the clinical value is underlined. Total metabolic tumor volume (TMTV), which represents the overall metabolic activity of the tumor, strongly correlates with baseline ctDNA concentration (r = 0.68, *p* < 0.001).^31^ Combining total metabolic tumor volume ≥ 220 cm^3^ and ctDNA ≥ 1.5% identifies high-risk cohorts with a 3-year cumulative DLBCL relapse rate of 82%.^32^

The integration of PET-CT radiomic features with ctDNA analysis has been demonstrated to construct superior prognostic models that outperform traditional clinical indices. A comprehensive study developed and validated a multi-parameter prognostic score by combining quantitative PET metrics with ctDNA levels and molecular clustering. In multivariate analysis, both high ctDNA levels (HR 2.04, 95% CI 1.10–3.78) and high-risk PET features (HR 3.84, 95% CI 1.78–8.28) independently predicted PFS.^33^ A two-factor model stratifying patients into low- (0 points), intermediate- (1–2 points), and high-risk (3 points) groups revealed starkly distinct 40-month PFS rates of 93.0%, 61.5%, and 33.6%, respectively (*p* < 0.001).^33^ The integration of the biological context provided by liquid biopsy further refined this model. Patients belonging to the favorable BN2, EZB, or ST2 molecular clusters exhibited significantly better outcomes.^33^ A subsequent three-factor prognostic model incorporating ctDNA levels, PET metrics, and molecular cluster assignment demonstrated even more precise risk stratification, with 40-month PFS rates of 91.1%, 58.1%, and 12.1% across risk groups (*p* < 0.001).^33^ Critically, this three-factor model achieved a higher C-index for both PFS and overall survival than the two-factor model and significantly outperformed the IPI and cell-of-origin classification in multivariate analysis, establishing a new paradigm for combining anatomical, metabolic, and molecular data for outcome prediction.^33^

Beyond mutation tracking, integrative biomarkers like the Programmed Cell Death Index (PCDI), which combines ctDNA-based apoptosis-related gene expression (e.g., BCL2, CASP8) with tumor burden, show utility in predicting responses to targeted therapies and correlate with ctDNA clearance rates (r = 0.62), facilitating dynamic treatment adjustments.^34^ In DLBCL patients treated with BCL2 inhibitors, those with high PCDI values achieved an objective response rate (ORR) of 75%, whereas the ORR was 29% in the low-PCDI cohort.^23^ A multicenter study in 2024 found that patients with less-defined subtype DLBCL and low expression of CASPA8 had a significantly decreased objective response rate to the venetoclax combination regimen (OR = 0.41, *p* = 0.002), and its mutation VAF was highly correlated with the clearance rate of ctDNA (r = 0.62, *p* = 0.004), demonstrating both predictive and dynamic monitoring value for therapeutic efficacy.^23^

Furthermore, high levels of ctDNA are closely associated with the poor prognosis of aggressive non-Hodgkin's lymphoma, with DLBCL having the most conclusive evidence. The median concentration of ctDNA in the plasma of newly diagnosed patients was significantly higher than that of healthy controls (13.7 ng/dL *vs* 7.4 ng/dL, *p* < 0.001), and high ctDNA significantly shortened both event-free survival (EFS) and overall survival (OS), with hazard ratios of 2.14 (95% CI 1.31–3.40) and 2.51 (95% CI 1.84–3.40), respectively, as independent prognostic factors independent of the IPI.^8^ The prospective POLARIX trial (*n* = 878) confirmed that the PFS and OS in the high ctDNA group were worse than those in the low ctDNA group, and were significantly correlated with IPI, LDH, and TMTV.^8^ A multicenter study further used a cutoff value of 2.5 log_10_ hGE/mL, finding that the 4-year PFS in the high ctDNA group was only 50.3%, significantly lower than 71.7% in the low ctDNA group (*p* = 0.0018), and raised the risk stratification C-statistic from 0.59 to 0.63^9^. Therefore, high ctDNA is not only a real-time mirror of tumor burden in DLBCL, but also a quantitative and independent adverse prognostic indicator, which has been consistently verified by many high-quality studies.

### MRD detection and recurrence prediction

The detection of MRD using ctDNA is paramount for predicting recurrence in DLBCL. Dynamic monitoring reveals that high-risk patients, often characterized by non-GCB subtype, advanced Ann Arbor stage, and high IPI scores, not only test positive for MRD but also exhibit significant fluctuations in ctDNA load.^35^ The prognostic power of ctDNA-MRD is most critical at the end-of-treatment (EOT) timepoint. Achieving MRD negativity is a powerful favorable indicator; a pooled analysis demonstrated that 100% of patients who were MRD-negative at EOT (*n* = 70) experienced no progression with a median follow-up of 17 months, whereas MRD positivity was associated with a drastically reduced 2-year overall survival (38% *vs* 93%).^35^ The kinetics of ctDNA clearance during therapy are also highly prognostic. While clearance after any of the first three treatment cycles is favorable for PFS, the failure to clear ctDNA by EOT defines the highest-risk group.^35^

Ultra-sensitive detection technologies are crucial for accurate MRD assessment. PhasED-Seq, which targets phased variants, has demonstrated exceptional analytical performance, with a false-positive rate of 0.24%, a limit of detection of 0.7 parts per million, and > 96% precision.^36^ This sensitivity is critical, as 38% of patients who ultimately relapse have ctDNA levels below the 0.01% threshold at EOT.^35^ In clinical validation, PhasED-seq achieved 100% sensitivity for identifying progression events, significantly outperforming PET/CT, which had a sensitivity of only 50% at matched specificity (91%).^35^

The superior sensitivity of ultra-deep ctDNA sequencing translates into a critical clinical advantage: the lead time for relapse detection. Studies have consistently shown that ctDNA-based MRD detection can identify molecular recurrence months before it becomes apparent on conventional imaging.^33^ In the context of CAR-T cell therapy, ctDNA detection preceded or coincided with radiographic recurrence in 94% of patients. For patients receiving frontline immunochemotherapy, PhasED-Seq demonstrated the ability to detect MRD up to 10 months before clinical recurrence, a window during which PET-CT remained negative.^33^ This lead time provides a potential therapeutic window for early intervention before overt, often treatment-resistant, disease manifests.^33^

The integration of mutational data from ctDNA further refines risk stratification and reveals heterogeneity in treatment resistance. Mutations associated with poor prognosis, such as CCND3, and those linked to drug resistance, like NSD2, are more prevalent in the plasma of high-risk patients.^35^ This molecular profiling complements the PCDI, which itself is associated with MRD status and other high-risk clinical features.^35^

Despite its promise, the clinical implementation of ctDNA for lymphoma MRD assessment faces challenges, including the need for rapid turnaround times, a lack of standardized clinically available assays, and variable performance across platforms.^37^ Initiatives like the ASH ctDNA Working Group aim to establish standards for pre-analytical processing and bioinformatics to harmonize the technology.^37^ Furthermore, high costs and accessibility concerns may limit widespread adoption.^38^^,^^39^ Nonetheless, the robust evidence solidifies ctDNA-MRD, particularly when assessed with ultra-sensitive methods like PhasED-Seq at EOT, as a highly relevant tool for risk stratification and a potential surrogate endpoint in clinical trials.^35^

### Clinical integration strategies

Modern adaptive clinical trial designs are harnessing real-time ctDNA dynamics to optimize therapeutic precision. In the POLARIX trial (NCT03274492), the Pola-R-CHP regimen demonstrated superior 2-year progression-free survival (76.7% *vs* 70.2%, HR = 0.73) in DLBCL, though its protocol did not incorporate ctDNA-guided therapy adjustments.^31^ Notably, polatuzumab vedotin-based regimens have been approved and implemented in clinical practice for DLBCL across different treatment stages: for relapsed/refractory (R/R) disease, regimens include pola-BR (polatuzumab vedotin + bendamustine + rituximab) and pola-rituximab; for frontline treatment, Pola-R-CHP (polatuzumab vedotin replacing vincristine in the R–CHOP backbone) has become a standard option. A key advantage of these regimens lies in their compatibility with ctDNA dynamic monitoring: plasma ctDNA tracking can identify molecular progression 6–8 weeks before radiological recurrence in patients receiving polatuzumab-containing regimens, providing a window for early therapeutic intervention.^40^^,^^41^ Additionally, Pola-R-CHP offers not only improved efficacy (2-year PFS 76.7% *vs* 70.2% with R–CHOP, HR = 0.73) but also reduced peripheral neuropathy—a common adverse event with vincristine—thus balancing efficacy and safety for comprehensive DLBCL management. In parallel, exploratory analyses from POLARIX revealed that early molecular responders (≥ 2.5 log-fold ctDNA reduction by Cycle 2 Day 1) had significantly improved 24-month outcomes (87.0% PFS *vs* 65.7%, HR = 2.89).^31^ This finding was further validated in the PharmaClon trial (NCT03190928), which showed that ctDNA clearance dynamically guided chemotherapy de-escalation or intensification within 1 treatment cycle.^32^ For suboptimal responders, rapid therapeutic escalation within 72 h—a strategy pioneered in KEYNOTE-789 (NCT03629431) for lung cancer—has shown survival benefits in ctDNA-defined high-risk solid tumors (HR = 0.62 for OS), suggesting its potential applicability to hematologic malignancies.^42^ Three clinically validated ctDNA thresholds now guide decision-making pathways as follows: Early molecular success (LFC ≥ 2.5): Maintain standard regimens like Pola-R-CHP; Inadequate clearance (LFC < 1.5): Escalate to R-ICE with 79% specificity for predicting 24-month failure; Intermediate response (LFC 1.5–2.5): Targeted intervention with BTK inhibitors, achieving AUROC 0.73 for risk stratification.^43^

This molecular-guided framework extends to staging refinement, where ctDNA mutation burden (≥ 5 variants/L) matches well with Ann Arbor classification (*κ* = 0.71).^22^ Prognostic models gain precision through TP53 mutation integration (C-index = 0.79), while clonal evolution analyses reveal that metastatic clones exceeding 30% frequency are associated with a significantly higher risk of progression (HR = 3.2)—a finding that is being prospectively validated in trials like SMART-DLBCL (NCT05283187), which dynamically adjust therapies based on ctDNA trajectories.^44^^,^^45^

### Resistance mapping via single cell ctDNA sequencing in DLBCL

ctDNA analysis provides critical molecular evidence to decipher drug resistance mechanisms in DLBCL. The CD20 protein encoded by the MS4A1 gene is the most classic transmembrane antigen on the surface of B cells and is also the direct target of anti-CD20 monoclonal antibodies such as rituximab. In DLBCL, MS4A1 mutations are not “primary’ driving events but are acquired resistance mechanisms that are “selectively enriched” under the pressure of R–CHOP or anti-CD20 monoclonal antibody treatment.^42^ In targeted therapy resistance, missense mutations in the transmembrane domain of the MS4A1 gene (e.g., Cys152Ser and Tyr158Asn) induce conformational changes in the CD20 protein through steric hindrance, reducing rituximab binding affinity by 5.3-fold. Longitudinal monitoring reveals clonal selection of these mutations during R–CHOP therapy, with VAFs increasing from 3.2% at baseline to 8.7% after three treatment cycles (2.7-fold). These mutations correlate significantly with reduced CD20 immunohistochemical staining intensity (Spearman *ρ* = −0.68), which suggests that antigen epitope loss may be a key mechanism of immunotherapy resistance.^42^

BCR signaling pathway reactivation represents another core resistance mechanism. The CD79B Y196H mutation disrupts the immunoreceptor tyrosine-based inhibitory motif (ITIM), which reduces ibrutinib-mediated apoptosis by 41%. The CARD11 L251P mutation constitutively activates the CBM complex, increasing NF-κB transcriptional activity by 4.1-fold. Additionally, high VAFs (> 25%) of the MYD88 L265P mutation predict CAR-T therapy failure (sensitivity 73%, specificity 88%, AUC = 0.82), mediated by TLR9-driven cytokine storms and T-cell exhaustion^46^, ^47^, ^48^.

Clonal evolution analysis identifies two resistance trajectories: accelerated proliferation of residual clones during chemotherapy intervals (Ki-67 index rising from 35% to 78%, *p =* 0.002) and post-CAR-T enrichment of TNFRSF14/GNA13 mutations (4.8-fold enrichment, *q* < 0.01). The latter drives immune evasion via PD-L1 up-regulation (RNA-seq differential expression 5.3-fold, FDR = 0.003). A multifactorial model incorporating GNA13 mutations (HR = 3.2), SOCS1 deletions (HR = 2.8), and IL6R amplifications (HR = 1.9) stratifies hyperprogression risk, distinguishing median progression-free survival as 2.1 *vs* 8.3 months.^48^^,^^49^

Beyond mapping resistance mechanisms, ctDNA dynamics serve as a powerful early-warning system for clinical outcomes following CAR-T cell therapy. In a prospective multi-institutional trial focusing on axi-cel for DLBCL, ctDNA monitoring demonstrated exceptional prognostic utility.^29^ The ability to achieve undetectable ctDNA levels as early as one-week post-infusion was strongly associated with durable responses (70% in durable responders *vs* 13% in patients with progression, *p* < 0.0001). In the Day 28 landmark analysis, the detection of ctDNA was a critical negative predictor, delineating patients with markedly inferior median progression-free survival (3 months *vs* not reached, *p* < 0.0001) and overall survival (19 months *vs* not reached, *p* = 0.0080). Notably, ctDNA analysis significantly refined response assessment in patients with indeterminate radiographic findings (partial response or stable disease), where its presence accurately identified those at high risk of imminent relapse. ctDNA detection preceded or coincided with radiographic recurrence in 94% of patients, underscoring its lead-time advantage. All patients with a durable response achieved undetectable ctDNA by 3 months post-infusion.^29^ Complementing these findings, independent work has validated specific ctDNA thresholds, showing that high pre-infusion levels and failure to achieve a major molecular response (MMR, defined as a 2.5-log10 reduction) by week 4 post-CAR T-cell infusion are robust determinants of treatment failure and shorter event-free survival.^30^

Epigenetic dysregulation also contributes to resistance. KMT2D mutations induce H3K4me3 loss at the BCL2 promoter (ChIP-seq *q* = 0.004), elevating BCL2 expression by 3.7-fold (*p =* 0.001) and conferring venetoclax resistance. The EZH2 Y646 mutation enhances DNMT3A target methylation (β-value 0.41 *vs* 0.19, *p =* 0.008), correlating with HDAC inhibitor resistance and shortened median overall survival (4.2 *vs* 11.5 months). SUZ12/EED mutations reduce lenalidomide binding affinity (Kd increased from 12 nM to 31 nM, *p =* 0.009), decreasing overall response rates from 68% to 29% (*p =* 0.003).^27^

### Multi-omics guided prognostic stratification in DLBCL

The integration of ctDNA with complementary biomarkers has catalyzed precision prognostication. Combining serum TARC (cut-off 800 pg/mL) with ctDNA burden (≥ 0.25%) stratified DLBCL into 3 prognostic groups: high-risk cohort showed a 2-year PFS of 31% (95% CI: 25%–37%) versus 89% in low-risk group (HR = 2.1, *p* < 0.001).^50^ This combinatorial approach mitigates the limitations of standalone ctDNA quantification, particularly in cases with low tumor shedding.

Dynamic biomarker profiling further enhances predictive power. Exosomal BCL2 mRNA monitoring revealed a < 50% decrease by cycle 2 associated with a 3.3-fold higher relapse risk (*p =* 0.004), outperforming ctDNA variant allele frequency (AUC 0.78 *vs* 0.65).^26^ The superior performance of exosomal RNA in detecting early treatment resistance suggests distinct biological information compared to ctDNA fragmentation patterns.

Translational impact is evidenced in therapeutic decision-making: This model increased the early intervention rate in high-risk patients from 22% to 58% within R–CHOP protocols (*p =* 0.002), demonstrating how multi-omics integration can transform static risk stratification into dynamic clinical action.

### Clinical translation challenges and solutions

#### *Technical limitations*

Current ctDNA assays often encounter false-positive results when detecting mutations with a VAF below 0.1%. To address this issue, recent advancements have introduced dual-index unique molecular identifiers combined with optimized hybridization probes, which significantly reduce the false-positive rate from 0.5% to 0.02%.^51^ Additionally, Baird et al demonstrated that further enhancing specificity to 99.8% can be achieved by increasing probe coverage to at least 98% and employing bioinformatics filtering techniques, such as excluding blacklist regions.^52^ These improvements are crucial for enhancing the reliability of low-frequency mutation detection.

Besides the false-positive issue in low-frequency mutation detection, ctDNA-related analysis also faces challenges from the instability of cell-free RNA. The inherent instability of cfRNA poses formidable standardization challenges, with RNase-mediated degradation (half-life < 2 h at room temperature) demanding stringent preanalytical protocols. Currently, guidelines clearly stipulate that immediate blood stabilization should be carried out using specialized tubes (e.g., Streck Cell-Free RNA BCT), followed by plasma processing within 4 h—a critical window to preserve transcriptomic integrity.^53^ Recent technological advances address these hurdles, as demonstrated by Yan et al's implementation of automated platforms like QIAcube, which boosts cfRNA recovery efficiency by 35% while ensuring consistent RNA quality (RIN ≥ 7) through standardized workflows.^54^

### Biological complexity

Clonal and spatial heterogeneity interference: Intra-DLBCL heterogeneity leads to significant variations in ctDNA abundance. For instance, there can be 100-fold differences in subclonal mutations. This phenomenon reduces the sensitivity of ctDNA detection to 67%.^43^ Stuckey et al proposes multi-region liquid biopsies (repeated sampling at 24-h intervals), increasing sensitivity to 92% (*p =* 0.03).^10^

Beyond the interference from clonal and spatial heterogeneity, another challenge lies in differentiating microenvironment-derived cfDNA. Stromal cell-derived cfDNA makes up 15%–40% of total cfDNA,^12^ and has confounding methylation features, such as LINE-1 hypomethylation. To address this, Wang et al developed an XGBoost-based machine learning classifier. With an AUC of 0.91, this classifier uses methylation fingerprints and validates mitochondrial DNA copy number as a discriminator, which can reduce the interference of non-tumor-derived cfDNA by 60%.^55^

### Clinical implementation barriers

One of the key challenges in clinical implementation is the lack of harmonization between ctDNA and imaging thresholds. Currently, the integration of ctDNA and PET/CT thresholds remains unresolved, which poses a significant hurdle for accurate prognostic stratification. To address this issue, the NCT04553406 trial has been initiated, aiming to enroll 500 DLBCL patients to evaluate a composite prognostic stratification model that combines ctDNA levels (≥ 0.1% VAF) with PET/CT findings (SUVmax ≥ 4).^56^ Additionally, Alig et al have proposed a dynamic threshold model, which uses a slope of ≥ 0.3 per cycle to predict radiological progression six weeks earlier than traditional methods (HR = 2.1, *p =* 0.01).^57^ This innovative approach could significantly improve the timeliness and accuracy of treatment response assessment.

In addition to the lack of harmonization in thresholds, the high cost of ctDNA testing also poses a significant obstacle to its widespread clinical implementation. ctDNA testing's global adoption faces significant economic hurdles. Currently, the cost of a single ctDNA test ranges from $1200 to $1800. Through optimized treatment algorithms that can reduce unnecessary therapies by 62%, the cost-effectiveness analysis shows that the cost per quality-adjusted life-year is still high, which may limit its widespread use.^58^^,^^59^ A phased insurance model prioritizing relapsed/refractory cases—with eventual expansion to frontline monitoring—could bridge cost-efficacy gaps. Regions adopting ctDNA-guided protocols achieve 15% higher 3-year lymphoma survival rates, underscoring the urgency for international collaboration to standardize cost-containment strategies without compromising test rigor.

It was not clear what the main challenges now are for the field or how they can be overcome. To clarify, the core challenges converge on three interconnected fronts: technical bottlenecks in ultra-low frequency mutation detection and ctDNA stability, biological confounders from clonal heterogeneity and non-tumor ctDNA interference, and clinical barriers, including unstandardized thresholds, high costs, and limited accessibility. Addressing these requires synergistic solutions: advancing assay sensitivity via dual-index UMI and optimized probes, developing AI-driven classifiers to mitigate biological noise, harmonizing ctDNA-imaging integration through large-scale trials like NCT04553406, and implementing phased insurance models to improve cost-effectiveness. Cross-sector collaboration between technologists, clinicians, and policymakers will be pivotal to standardize protocols, reduce costs, and translate ctDNA's potential into routine clinical benefit for DLBCL patients.

## Conclusion

In conclusion, ctDNA has emerged as a powerful tool in the management of DLBCL, directly addressing the challenges presented by the disease's variability and the limitations of traditional monitoring strategies, as initially introduced.

Technologically, the evolution of ctDNA detection and multi-omics integration has been remarkable. Advanced platforms and profiling techniques have enhanced our ability to detect mutations, trace ctDNA origins, and distinguish tumor-specific signals. This has led to more accurate early diagnosis, molecular subtyping, and prognostic evaluation. Clinically, ctDNA plays a crucial role in treatment response monitoring and resistance mechanism analysis in DLBCL. It can precisely reflect treatment efficacy during therapies like R–CHOP or CAR-T and predict relapse. Additionally, it helps in understanding resistance mechanisms and is valuable in post - transplant monitoring.

Despite the significant progress made in ctDNA detection and its applications in DLBCL management, several challenges remain. First, technical limitations, such as the detection of low-frequency mutations and the instability of ctDNA, need to be addressed through the development of more sensitive and robust assays. Second, the biological complexities of DLBCL, including clonal and spatial heterogeneity, require advanced analytical methods to accurately interpret ctDNA data. Finally, the high cost of ctDNA testing poses a significant barrier to its widespread clinical adoption. To overcome these challenges, it is essential to standardize protocols across different platforms, drive technological innovation to improve sensitivity and reduce costs, and foster multidisciplinary collaboration to integrate ctDNA-based approaches into routine clinical practice. By addressing these issues, we can offer more personalized and effective treatment options for DLBCL patients, ultimately improving their outcomes and quality of life.

## CRediT authorship contribution statement

**Ruoyun He:** Writing – original draft. **Wanyue Zhang:** Writing – original draft. **Lisha Li:** Writing – review & editing. **Lintao Bi:** Writing – review & editing, Validation, Supervision, Project administration, Funding acquisition.

## Funding

This work was funded by the 10.13039/501100009991Department of Finance of Jilin Province (No. 2024SCZ58).

## Conflict of interests

The authors declare that they have no conflict of interests.
